# Pathway economy in cyclization of 1*,n*-enynes

**DOI:** 10.3762/bjoc.21.173

**Published:** 2025-10-27

**Authors:** Hezhen Han, Wenjie Mao, Bin Lin, Maosheng Cheng, Lu Yang, Yongxiang Liu

**Affiliations:** 1 Key Laboratory of Structure-Based Drug Design and Discovery (Shenyang Pharmaceutical University), Ministry of Education, Shenyang 110016, P. R. China; 2 Wuya College of Innovation, Shenyang Pharmaceutical University, Shenyang 110016, P. R. China; 3 Institute of Drug Research in Medicine Capital of China, Benxi 117000, P. R. China

**Keywords:** economical synthesis, 1,*n*-enynes, pathway economy, small-molecule skeletons

## Abstract

This review presents a paradigm-shifting "pathway economy" strategy for 1,*n*-enyne cyclization, enabling divergent construction of complex molecular architectures from a single substrate class. Through mechanistic-guided modulation of catalysts, solvents, ligands, and angle strain, this approach achieves unprecedented reaction pathway control while demonstrating superior temporal and step efficiency compared to conventional methods. The work establishes a sustainable framework for rapid molecular diversification, offering transformative potential for green chemistry and pharmaceutical applications. By unifying mechanistic insights with practical synthetic design, this review provides valuable guidance for future innovations in precision organic synthesis.

## Introduction

As organic synthesis concepts continue to evolve, economical synthesis remains a foundational principle for synthetic chemists [1–7]. The essence of economical synthesis lies in the conservation of materials and time, thereby facilitating the synthesis of target molecules at lower cost while minimizing environmental pollution through minimized waste generation.

**Scheme 1:**
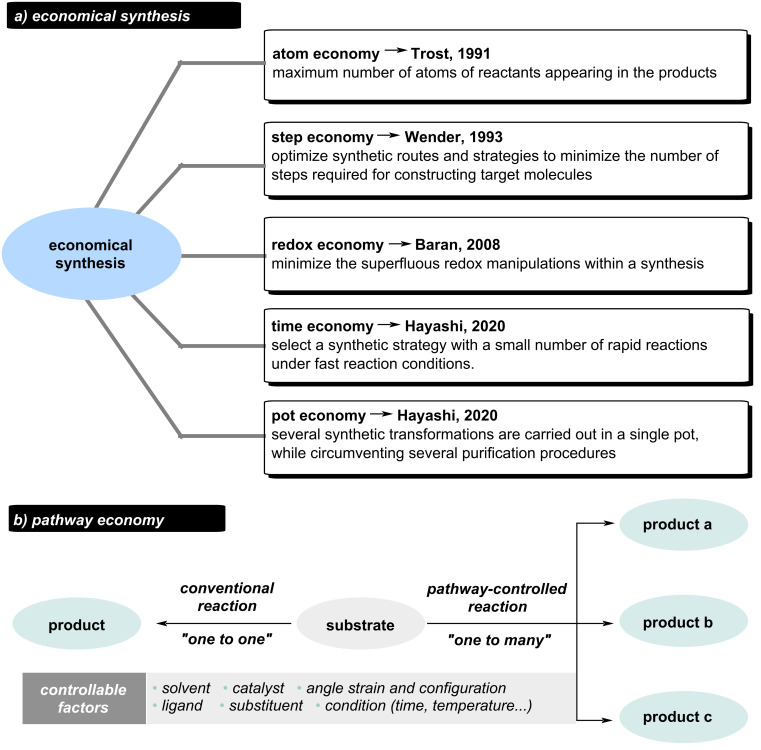
Economical synthesis and pathway economy.

In 1991, Trost first introduced the concept of “atom economy”, proposing that an ideal reaction should incorporate all atoms of the reactants, thereby enabling more efficient utilization of limited raw materials [1–2]. Two years later, Wender proposed “step economy” advocating optimized synthetic routes and strategies to minimize the number of steps required for constructing target molecules [3–4]. In 2008, “redox economy” was introduced by Baran [5]. Similar to “atom and step economies”, this concept emphasizes prioritizing the minimization of redox manipulations during synthesis to achieve linear and stable progression of oxidation states in intermediates. In recent years, “pot economy” and “time economy” were proposed by Hayashi, underscoring the importance of reducing the time required for reaction processes and conducting multistep reactions within a single pot (Scheme 1a) [6–7].

Contemporary organic synthesis is progressively approaching ideal synthesis – achieving highly functionalized target molecular frameworks in a single step. This paradigm circumvents cumbersome late-stage functional group manipulations and perfectly aligns with the principles of step economy, time economy, and redox economy.

Over the past few decades, chemists have rapidly directed this efficient synthetic paradigm toward diverse targets by skillfully regulating reaction components such as solvents, catalysts, ligands and so on. Unlike traditional “one-to-one” reactions, pathway-controlled “one-to-many” transformations synthesize multiple products from single intermediates, dramatically reducing preparation time and reagent requirements (Scheme 1b). As an exceptionally significant reaction, 1,*n*-enyne cyclization is capable of constructing a variety of complex small-molecule frameworks, including fused and bridged rings, thereby serving as a potent tool in the syntheses of natural products and pharmaceuticals. Our group has pioneered the concept of "pathway economy" by systematically regulating reaction parameters (solvent, substituent, ligand, catalyst) in 1,*n*-enyne cyclizations, enabling efficient "one-to-many" transformations that drastically reduce synthetic steps and resource consumption.

## Review

### Solvent-controlled cyclization of 1,*n*-enynes

**Scheme 2:**
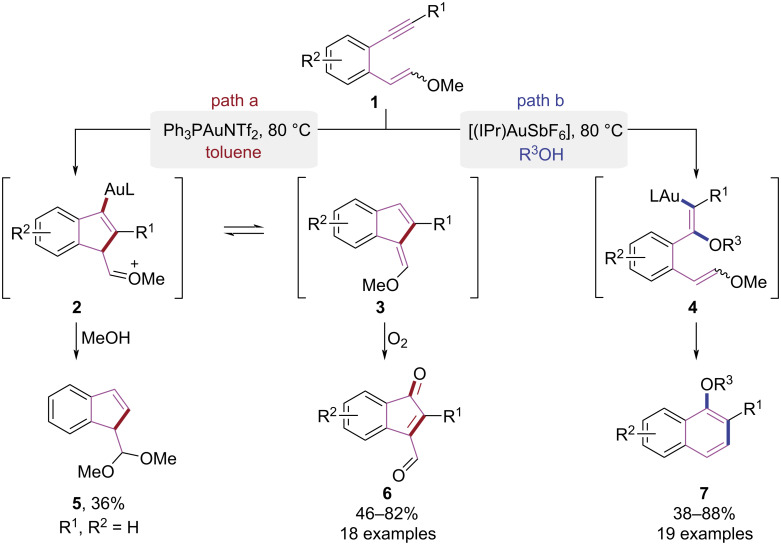
Au(I)-catalyzed cascade cyclization paths of 1,5-enynes.

Solvents play a multifaceted regulatory role in chemical transformations, exerting kinetic modulation through solvation effects on activation barriers and reaction rates, dictating thermodynamic equilibria that govern product distribution, and enabling precise reaction pathway regulation via selective stabilization of critical transition states. This integrated control framework provides a rational basis for designing reaction conditions to optimize selectivity and efficiency in organic synthesis. In 2014, the Liu group developed an Au(I)-catalyzed cascade cyclization strategy for synthesizing polysubstituted naphthalenes using 1,5-enynes **1** as substrates involving alkyne alkoxylation and dienol ether aromaticity-driven processes (Scheme 2) [8]. The reaction pathway was decisively influenced by the choice of solvent. Under gold catalysis, with toluene as the solvent and 2 equiv of methanol serving as the nucleophile, the reaction proceeded via 5-*endo*-dig cyclization. This pathway involved enol ether attack on the gold-activated alkyne, leading to the formation of oxonium intermediate **2**. Subsequently, nucleophilic addition of methanol culminated in the formation of indene motif **5** (Scheme 2, path a). When methanol served dual roles as solvent and nucleophile, the gold-catalyzed intermolecular Markovnikov addition of methanol to the gold-activated alkyne proceeded to afford dienol intermediate **4**. The intermediate **4** subsequently underwent a regioselective 6-*endo-*trig cyclization, generating the naphthalene core **7** (Scheme 2, path b). In the following years, Liu and co-workers discovered that the protonation of intermediate **2** triggered its conversion to intermediate **3**, which subsequently underwent oxidation with oxygen, resulting in the generation of an indenone skeleton **6** [9]. This tunability achieved efficient and regioselective syntheses of indene, indenone, and naphthalene derivatives from simple aromatic 1,5-enyne substrates.

**Scheme 3:**
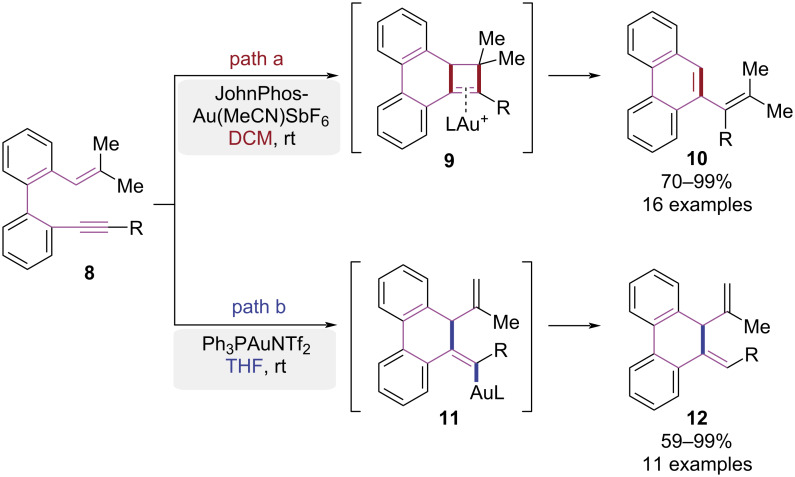
Au(I)-catalyzed cyclization paths of 1,7-enynes.

In 2020, a solvent-controlled strategy for Au(I)-catalyzed divergent syntheses of phenanthrene and dihydrophenanthrene derivatives was developed by the Rodríguez group (Scheme 3) [10]. In dichloromethane (DCM), gold(I)-catalyzed alkyne activation initiated 6-*endo*-dig cyclization of the conjugated alkene. Subsequent alkyl migration formed four-membered ring intermediate **9**, which underwent fragmentation and rearrangement to yield phenanthrene derivative **10** (Scheme 3, path a). When tetrahydrofuran (THF) was used as solvent, the proton elimination and protodeauration led to the formation of dihydrophenanthrene **12** due to solvent effects (Scheme 3, path b). This pathway-controlled approach for the syntheses of phenanthrenes complemented the established protocols.

**Scheme 4:**
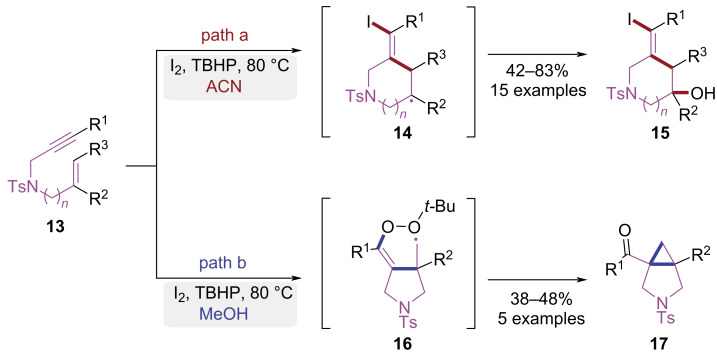
I_2_/TBHP-mediated radical cycloisomerization paths of 1,*n*-enyne.

In 2020, Mutra et al. achieved a radical initiated intramolecular cascade cyclization of 1,*n*-enynes to provide structurally diverse heterocycles (Scheme 4) [11]. Solvent selection dictated divergent reaction pathways under I_2_/TBHP oxidation. When an acetonitrile/water mixed solvent was used, iodine radical addition to the alkyne preferentially initiated 6-*endo*-trig cyclization, affording iodinated homoallylic alcohol piperidines **15** (Scheme 4, path a). Conversely, cyclopropane-annulated pyrrolidines **17** were constructed using methanol as solvent through hydroxyl radical-mediated 5-*exo*-trig cyclization pathway (Scheme 4, path b). This metal-free methodology delivered synthetically versatile iodinated homoallylic alcohols bearing piperidine motifs and pyrrolidine-fused cyclopropanes. Importantly, the reaction was carried out with high operational simplicity and environmental compatibility under mild reaction conditions.

**Scheme 5:**
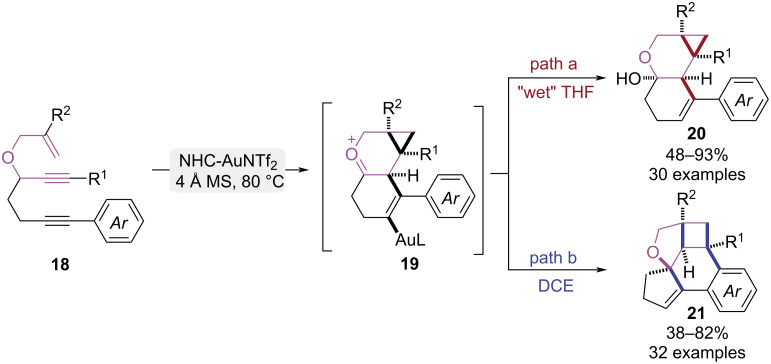
Au(I)-catalyzed cycloisomerization paths of 3-allyloxy-1,6-diynes.

In 2024, Chan and co-workers achieved an innovative gold-catalyzed cascade cycloisomerization of 3-allyloxy-1,6-diynes to access cyclopropane- and cyclobutane-fused benzofurans/chromanols (Scheme 5) [12]. In this study, solvent polarity and trace water were identified as key parameters governing the reaction pathway. In THF with trace water, water served as a nucleophile that participated in the reaction, triggering hydroxylation of cyclopropanation intermediate **19** and affording cyclopropane-fused chromanol products **20** (Scheme 5, path a). In anhydrous 1,2-dichloroethane (DCE), gold(I)-catalyzed cyclopropanation of 1,6-enyne initiated a cascade involving 1,5-enyne addition, consecutive 1,2-alkyl migrations, and Friedel–Crafts alkylation, efficiently constructing the pentacyclic fused benzofuran framework **21** (Scheme 5, path b). Above two analogues were prepared on a gram scale, converted into valuable synthetic intermediates, and used to successfully modify diverse bioactive scaffolds via late-stage functionalization, collectively demonstrating the method’s synthetic utility.

**Scheme 6:**
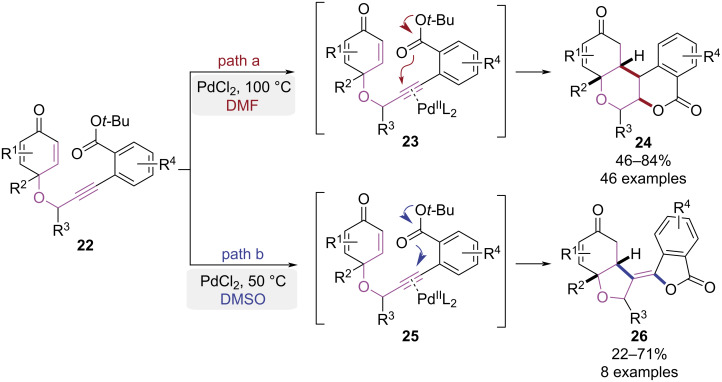
Pd(II)-catalyzed cycloisomerization paths of 2-alkynylbenzoate-cyclohexadienone.

In 2025, the Das group developed a palladium-catalyzed cycloisomerization of 2-alkynylbenzoate-cyclohexadienone that enables solvent-controlled selective syntheses of two polycyclic compounds (Scheme 6) [13]. Using PdCl_2_ as the catalyst and DMF as the solvent, substrate **22** underwent a 6-*endo*-dig cyclization and subsequent enone insertion, forming a palladium–carbon bond intermediate. Protonolysis yielded isocoumarin-fused dihydrochromenone skeleton **24** (Scheme 6, path a). When DMSO was employed instead, the strongly coordinating solvent diverted the reaction towards 5-*exo-*dig cyclization, furnishing a *Z*-configured tetrasubstituted alkene product **26** (Scheme 6, path b). The isocoumarin-fused dihydrochromenones prepared by this strategy could be further derivatized (e.g., via borylation, epoxidation), establishing a versatile platform for accessing fused-ring natural products.

### Substituent-controlled cyclization of 1,*n*-enynes

In transition metal-catalyzed cyclization reactions, the electronic properties and steric hindrance of substituents serve as critical switches for determining reaction pathway selection. Chemists have developed sophisticated synthetic strategies for programmable assembly of complex small molecular framework by leveraging substituent electronic and steric effects.

**Scheme 7:**
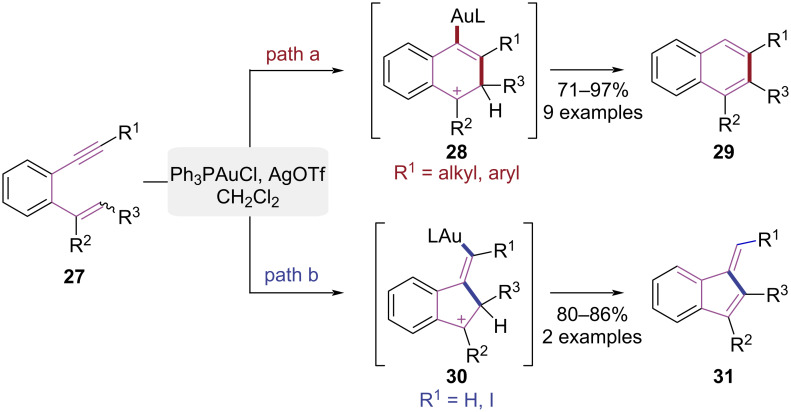
Stereoselective cyclization of 1,5-enynes.

In 2006, the Shibata group reported a gold-catalyzed cyclization approach using aromatic enyne derivatives, where substituent control governed the stereoselective syntheses of naphthalene and indene cores (Scheme 7) [14]. When the alkyne terminus of the substrate **27** bore an alkyl or aryl substituent, the Ph_3_PAuCl/AgOTf-catalyzed 6-*endo*-dig cyclization and subsequent deprotonation furnished 1,3-disubstituted or 1,2,3-trisubstituted naphthalenes **29** (Scheme 7, path a). When the alkyne terminus was iodo-substituted or unsubstituted, the 5-*exo*-dig cyclization pathway proceeded via selective activation of the iodoalkyne, generating 1-methyleneindene derivatives **31** (Scheme 7, path b). This work provided a novel approach for constructing substituted naphthalene and indene frameworks via gold-catalyzed cycloisomerization of 1,5-enynes.

**Scheme 8:**
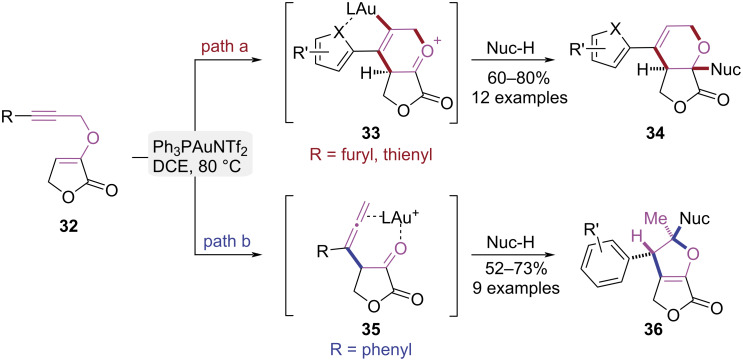
Substituent-controlled cycloisomerization of propargyl vinyl ethers.

In 2016, Liu et al. achieved the stereoselective syntheses of furofuran and furopyran scaffolds from propargyl vinyl ethers under gold(I) catalysis through a substituent-controlled strategy (Scheme 8) [15]. Substrates with heteroaryl substituents underwent 6-*endo-*dig cyclization via gold-heteroatom coordination, furnishing the lactone-fused pyran scaffold **34** (Scheme 8, path a). Substrates with aryl substituents at the terminal alkyne proceeded via a gold(I)-catalyzed propargyl-Claisen rearrangement, generating a β-allenic intermediate **35**. This intermediate underwent a Markovnikov-type nucleophilic addition followed by a 5-*exo-*trig cyclization to stereoselectively construct the furo[3,2-*b*]furan bicyclic framework **36** (Scheme 8, path b). The substituent-controlled gold(I)-catalyzed cycloisomerization of propargyl vinyl ethers provides a highly efficient platform for the divergent synthesis of functionalized furofuran and furopyran. Meanwhile, the preliminary antifungal assessment of these compounds underscored the synthetic value of this method.

**Scheme 9:**
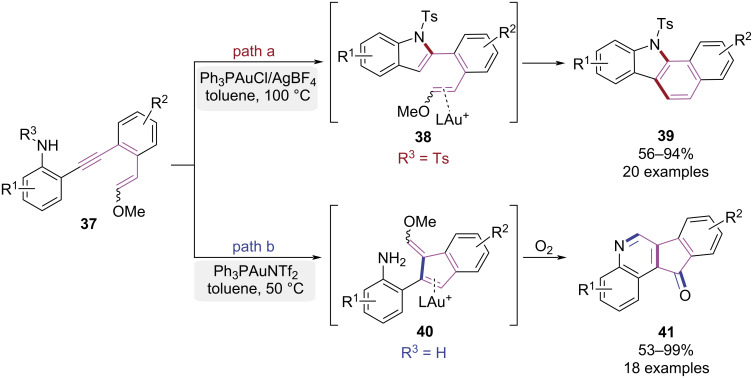
Au(I)-catalyzed pathway-controlled domino cyclization of 1,2-diphenylethynes.

In 2017, the Liu group established a precise control over cyclization sequences of 1,2-diphenylacetylenes by modulating the nitrogen-substitution patterns, enabling divergent syntheses of benzo[*a*]carbazole and indeno[1,2-*c*]quinoline derivatives (Scheme 9) [16]. When the nitrogen atom was substituted with strong electron-withdrawing groups, a nucleophilic attack to gold(I)-activated alkyne generated intermediate **38**, with subsequent 6-*endo*-trig cyclization affording benzo[*a*]carbazole **39** (Scheme 9, path a). Conversely, the activated alkyne was attacked by enol ether to yield intermediate **40** when using substrates with unsubstituted nitrogen. Concurrent oxygen-involved cyclization then furnished indeno[1,2-*c*]quinoline **41** (Scheme 9, path b). This work established a pathway-controlled strategy for efficient access to benzo[*a*]carbazole and indeno[1,2-*c*]quinoline derivatives.

**Scheme 10:**
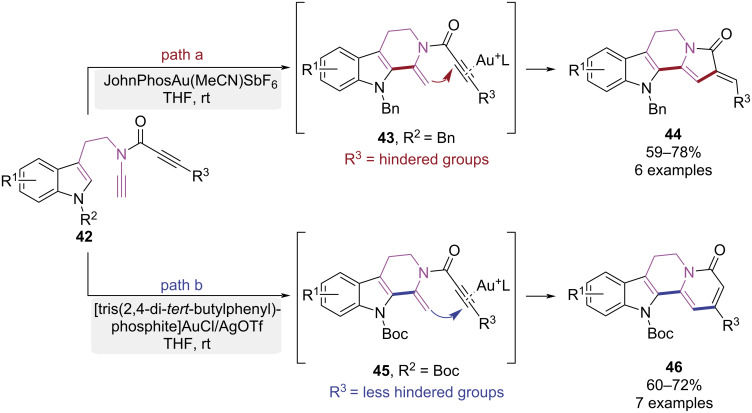
Au(I)-catalyzed tandem cyclo-isomerization of tryptamine-*N*-ethynylpropiolamide.

In 2019, the Liu group developed a gold-catalyzed tandem cycloisomerization, offering controllable synthesis of either indolo[2,3-*a*]quinolizine or indolizino[8,7-*b*]indole derivatives (Scheme 10) [17]. When the indole nitrogen of the substrate was substituted with electron-donating groups (EDGs) and the alkyne of propiolamide was equipped with a bulky substituent, the 6-*exo-*dig cyclization was initially triggered under gold(I)-catalysis, leading to intermediate **43**. Then the indolizino[8,7-*b*]indole skeleton **44** ultimately was constructed via a tandem 5-*exo-*dig cyclization (Scheme 10, path a). When the indole nitrogen was strategically functionalized with an electron-withdrawing groups (EWGs) and the alkyne of propiolamide was modified with a less bulky substituent. A 6-*exo-*dig cyclization yielded intermediate **45**. Finally, a tandem 6-*endo-*dig cyclization enabled the successful assembly of the indolo[2,3-*a*]quinolizine framework **46** (Scheme 10, path b). This controllable approach provided an efficient synthetic pathway for the related natural products.

**Scheme 11:**
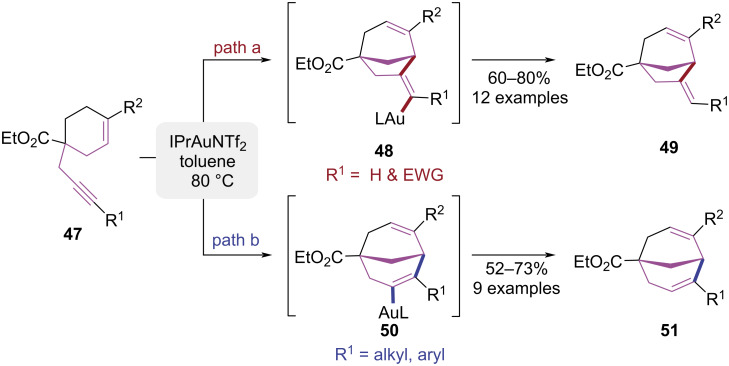
Au(I)-catalyzed tunable cyclization of 1,6-cyclohexenylalkyne.

In 2020, a gold-catalyzed divergent synthesis of bicyclo[3.2.1]oct-2-ene and bicyclo[3.3.1]nonadiene derivatives from 1,6-cyclohexenynes was reported by Davenel et al. (Scheme 11) [18]. The pathway selectivity was regulated by modulating substituents on the terminal alkyne. When the alkyne was unsubstituted, the vinylidene intermediate **48** was generated via a 5-*exo-*dig cyclization, which subsequently underwent protonolysis to yield the bicyclo[3.2.1]oct-2-ene product **49** (Scheme 11, path a). When aryl or alkyl substituents were introduced on the alkyne moiety, a more stable vinylidene intermediate **50** was formed via a 6-*endo-*dig cyclization, ultimately leading to the generation of the bicyclo[3.3.1]nonadiene product **51** (Scheme 11, path b). DFT calculations confirmed that the cyclization pathways were controlled by the influence of substituents on the stability of the intermediates. This work expanded the scope of gold-catalyzed reactions through systematic cycloisomerization studies of 1,6-enynes and an ethyl 4-oxocyclohexanecarboxylate-derived 1,7-enyne.

**Scheme 12:**
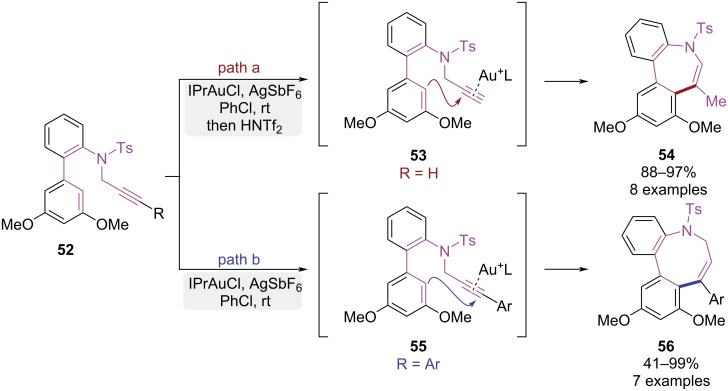
Substituent-controlled 7-*exo*- and 8-*endo*-dig-selective cyclization of 2-propargylaminobiphenyl derivatives.

In 2021, the Shibata group reported a gold-catalyzed ring isomerization strategy to synthesize medium-sized nitrogen heterocycles (Scheme 12) [19]. The seven-membered dibenzo[*b*,*d*]azepine and eight-membered dibenzo[*b*,*d*]azocine frameworks were successfully obtained through the modulation of alkyne terminal substituents. Under the catalysis of IPrAuCl/AgSbF₆, substrates bearing unsubstituted terminal alkynes underwent 7-*exo*-dig cyclization driven by the strong nucleophilicity of the 3,5-dimethoxyphenyl group, exclusively affording dibenzo[*b*,*d*]azepine **54** (Scheme 12, path a). Conversely, substrates with aryl-substituted internal alkynes underwent exclusive 8-*endo*-dig cyclization, efficiently delivering the strained dibenzo[*b*,*d*]azocine **56** (Scheme 12, path b). Distinct activation modes governed the selectivity, where regioselective terminal gold coordination triggered 7-*exo*-dig cyclization in terminal alkynes, whereas internal alkynes favored the 8-*endo*-dig pathway due to steric constraints and carbocation stability. This strategy facilitated efficient and selective synthesis of macrocyclic amines, with precise ring size control via substituent modulation.

**Scheme 13:**
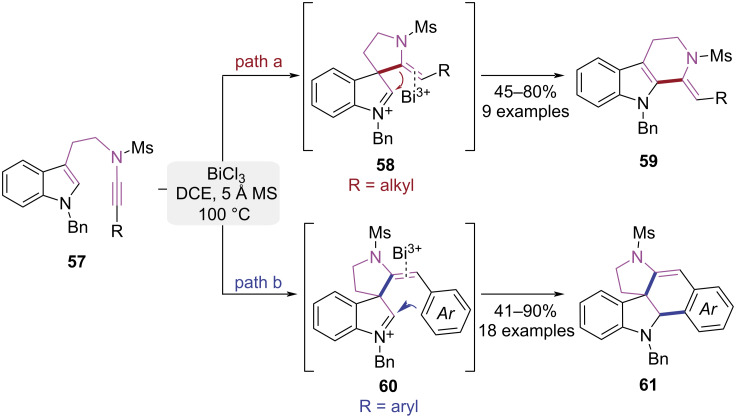
BiCl_3_-catalyzed cycloisomerization of tryptamine-ynamide derivatives.

In 2021, Liu group reported a BiCl_3_-mediated controllable cyclization of tryptamine-derived ynamides to synthesize two types of indole alkaloid skeletons (Scheme 13) [20]. For alkyl-substituted alkynes, the ynamide activated by BiCl_3_ was attacked by indole's C3-position to form spirocyclic intermediate **58**. Subsequent 1,2-migration then exclusively delivered tricyclic indole derivative **59** featuring an exocyclic *Z*-alkene (Scheme 13, path a). When the terminal substituent of the alkyne was an aryl group, C3-selective cyclization was triggered under BiCl_3_ catalysis to generate tricyclic iminium **60**. Subsequent aryl-assisted Mannich cyclization efficiently assembled the pentacyclic spiroindole framework **61** (Scheme 13, path b). The controllability of this cyclization process arose from the steric and electronic effects of the aryl group, where the π–π interactions and rigid structure of the aryl group facilitated the stabilization of the five-membered spiro ring. This work presents an efficient synthetic approach for two structurally complex classes of pentacyclic spiroindolines and tricyclic indoles of pharmacological significance.

**Scheme 14:**
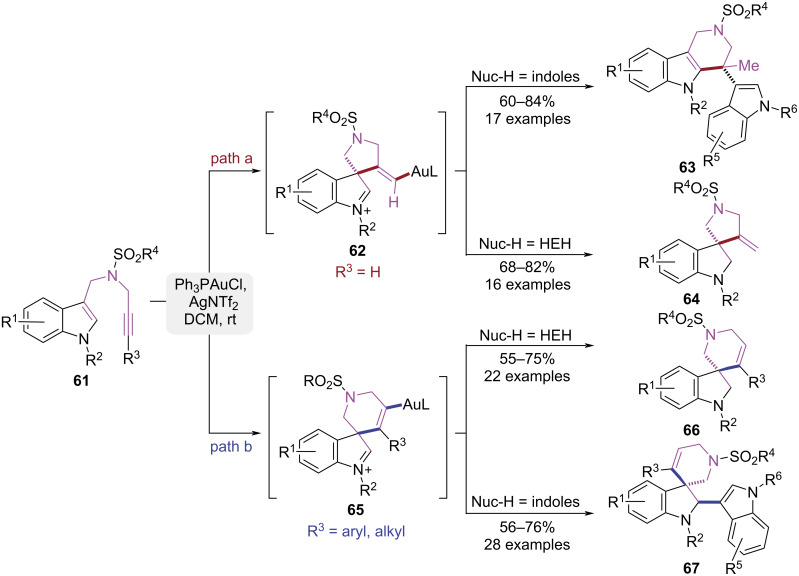
Au(I)-mediated substituent-controlled cycloisomerization of 1,6-enynes.

In 2023, the Liu group reported a controllable cyclization strategy for indole substrates featuring a 1,6-enyne motif, which afforded selective access to four indole derivatives through modulation of the alkyne's terminal substituents and nucleophile type (Scheme 14) [21–22]. The gold(I) catalyst activated the unsubstituted terminal alkynes to initiate a 5-*exo-*dig cyclization, generating a spiro[indoline-3,3*'*-pyrrolidine] intermediate **62** (Scheme 14, path a). When a less nucleophilic indole was used as the nucleophile, a Wagner–Meerwein rearrangement occurred, leading to the formation of a pyrido[4,3-*b*]indole product **63**. When Hantzsch ester (HEH) was used as the nucleophile, the imine intermediate **62** was reduced to product **64**. In contrast, when the alkyne was substituted with a phenyl group, the reaction shifted toward a 6-*endo-*dig cyclization pathway due to steric and electronic effects (Scheme 14, path b). When HEH was employed as the nucleophile, a spiro[indoline-3,3*'*-piperidine] framework **66** was formed. When indole acted as nucleophile, the stable imine–gold–aryl cation–π–π interaction precluded rearrangement and promoted the capture of imine to form spiro[indoline-3,3*'*-pyridine] derivatives **67**. The Ph_3_PAuCl/AgNTf_2_-catalyzed cyclization of *N*-propargyl-tethered amide enynes efficiently afforded four distinct heterocyclic scaffolds, which established a versatile platform for synthesizing structurally diverse indoline frameworks.

### Ligand-controlled cyclization of 1,*n*-enynes

**Scheme 15:**
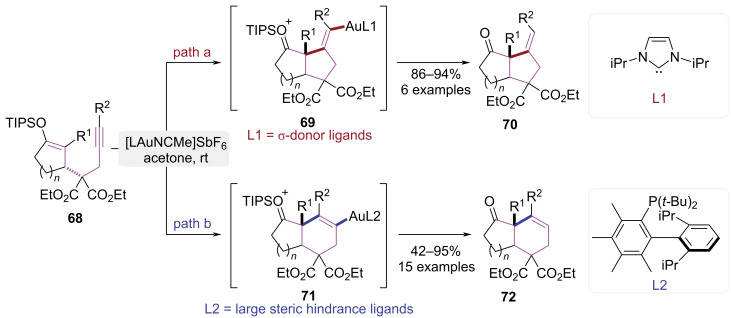
Ligand-controlled regioselective cyclization of 1,6-enynes.

The core function of catalyst ligands lies in their ability to precisely modulate catalyst performance through electronic and steric effects. The ligands could enhance catalytic activity and efficiency while enabling fine control over chemo-, regio-, stereo-, and enantioselectivity of reactions. Ligands enhance both the thermal/chemical stability and solubility profiles of catalysts in specific reaction media. Thus, ligands function as a regulatory nexus for the rational design and optimization of highly effective, selective homogeneous catalytic systems. In 2013, Barriault and co-workers demonstrated that strategic modulation of steric and electronic ligand parameters within gold(I)-catalyzed cyclization pathways enables the selective assembly of polycyclic aromatic heterocycles (Scheme 15) [23]. Catalysts bearing the strong σ-donating IPr ligand exhibited a marked preference for the 5-*exo*-dig cyclization pathway, affording five-membered ring product **70** (Scheme 15, path a). When a bulky Me_4_XPhos ligand was employed, the reaction favored 6-*endo-*dig cyclization, yielding a six-membered ring product **72** (Scheme 15, path b). This observed selectivity arose from differential stabilization of transition states by ligands, thereby enabling the Au(I)-catalyzed cyclization to directly access bioactive heterocyclic frameworks commonly encountered in natural products and pharmaceutical agents.

**Scheme 16:**
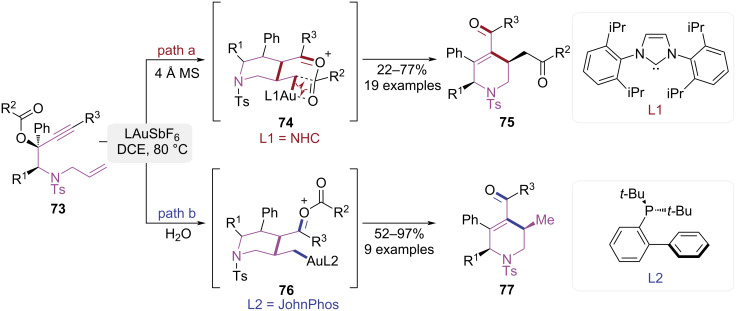
Ligand-dependent cycloisomerization of 1,7-enyne esters.

In 2013, Chan and co-workers reported a ligand-controlled cycloisomerization of 1,7-enyne esters affording the selective synthesis of *cis*-tetrahydropyridinones and δ-diketones (Scheme 16) [24]. When NHC-ligated gold catalysts were employed, a cascade sequence comprising 1,3-acyloxy migration, 6-*exo-*trig cyclization, and 1,5-acyl migration proceeded, affording δ-diketone-substituted *cis*-1,2,3,6-tetrahydropyridine derivatives **75** (Scheme 16, path a). The phosphine-ligated catalysts promoted an alternative pathway cascade involving 1,3-acyloxy migration, 6-*exo-*trig cyclization, and hydrolysis, exclusively producing *cis*-tetrahydropyridin-4-one derivatives **77** (Scheme 16, path b). Mechanistic studies revealed that intermediate **74** could be stabilized by NHC ligands to facilitate 1,5-acyl migration, whereas phosphine ligands could accelerate proton dissociation in intermediate **76** to drive hydrolysis. This methodology tolerates structurally diverse 1,7-enyne esters and generates defined *cis*-1,2,3,6-tetrahydropyridine scaffolds, which serve as synthetic intermediates for complex natural products and pharmacologically relevant heterocyclic frameworks.

**Scheme 17:**
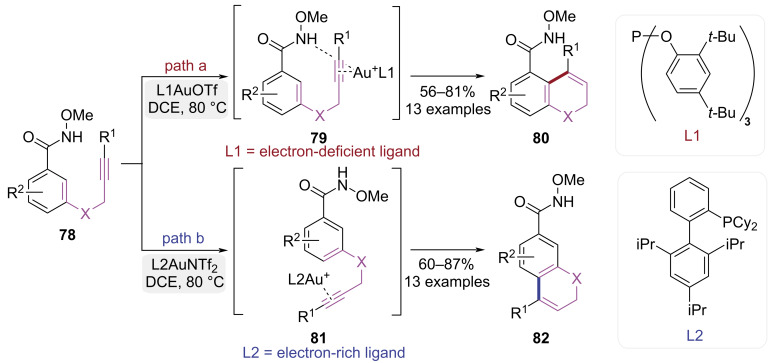
Ligand-controlled cycloisomerization of 1,5-enynes.

In 2016, Jiang group achieved *ortho*- and *para*-selective cyclization of methoxyamide-functionalized alkynes via ligand-controlled steric and electronic modifications (Scheme 17) [25]. When a flexible electron-deficient phosphate ligand **L1** was utilized, the Au(I)-catalyzed cyclization of substrates **78** resulted in the formation of *ortho*-cyclized products **80**, enabled by coordination to both the directing group and alkyne (Scheme 17, path a). However, the rigid electron-rich XPhos ligand (**L2**) promoted *para*-cyclization due to steric constraints and π–π stacking to yield dihydroquinoline derivatives **82** (Scheme 17, path b). This ligand-controlled Au(I)-catalyzed intramolecular hydroarylation overcame key challenges of poor regioselectivity and limited applicability to electron-deficient substrates.

**Scheme 18:**
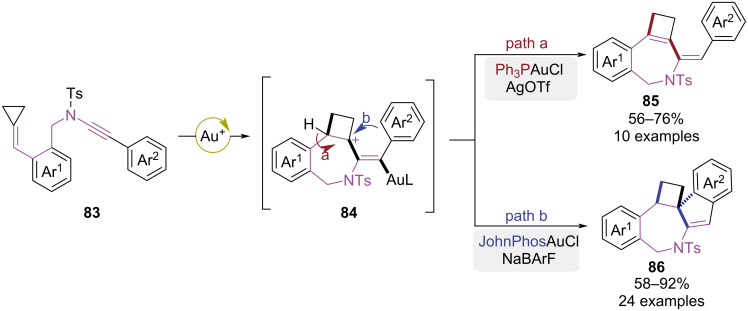
Ligand-controlled cyclization strategy of alkynylamide tethered alkylidenecyclopropanes.

In 2018, a gold-catalyzed divergent cyclization to access heteropolycyclic frameworks was reported by the Shi group (Scheme 18) [26]. Ph_3_PAuCl/AgOTf catalyzed a tandem 7-*exo*-dig cyclization followed by a cyclobutyl ring expansion process, yielding intermediate **84**. The deprotonation followed by protonolysis-mediated gold elimination delivered the ring-expanded product **85** (Scheme 18, path a). When a bulkier ligand was used, steric constraints promoted intramolecular Friedel–Crafts cyclization via intermediate **84** to form spiro-polycycle **86** (Scheme 18, path b). The ligand-dependent gold(I)-catalyzed cyclization provided modular access to therapeutically significant fused polycyclic heterocyclic scaffolds via regioselective ring expansion/cycloisomerization sequences.

**Scheme 19:**
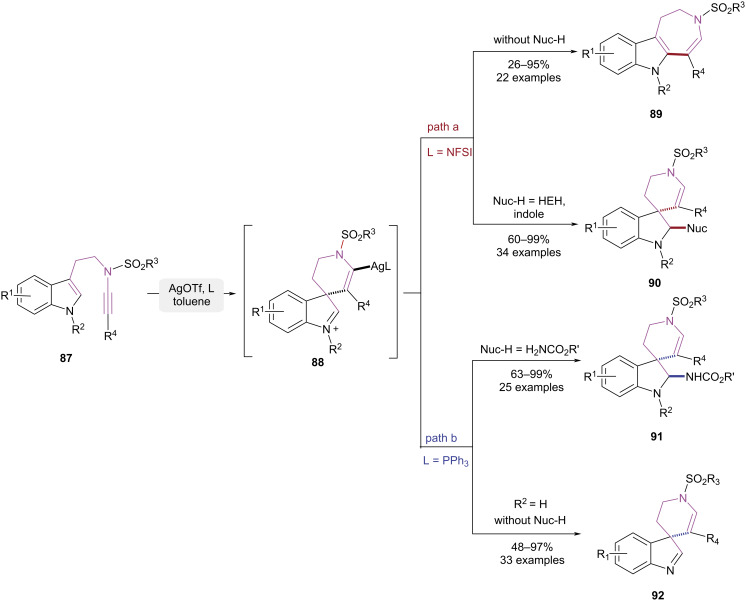
Ag(I)-mediated pathway-controlled cycloisomerization of tryptamine-ynamides.

In 2019 and 2020, Liu and co-workers achieved pathway-controlled cyclization–isomerization of tryptamine-ynamides using ligand-influenced silver catalytic systems (Scheme 19) [27–29]. To circumvent decomposition caused by the inherent high reactivity of ynamides under catalytic conditions, the authors attenuated the silver catalyst’s activity through ligand addition. This allowed for a umpolung addition of the substrate **87**, affording six-membered spirocyclic intermediate **88**. When the NFSI was used as ligand without nucleophiles, the tricyclic azepinoindole **89** was obtained via a Wagner–Meerwein rearrangement from the six-membered spirocyclic intermediate **88**. The addition of Hantzsch ester or indole as the nucleophile effectively trapped the imine intermediate **88**, preventing Wagner–Meerwein rearrangement and yielding spirocyclic indole framework **90** (Scheme 19, path a). When PPh_3_ was used as the ligand and amides as nucleophiles, the spirocyclic indole framework **91** was obtained via the capture of imine intermediate **88**. When the PPh_3_ was used as ligand in the absence of nucleophiles, the substrate **87** with an unprotected indole nitrogen facilitated the formation of intermediate **88**, enabling its stable isolation and subsequent affordance of the spirocyclic indole-derived imine product **92** (Scheme 19, path b). The suppression of Wagner–Meerwein rearrangement was attributed to the lower Lewis acidity of PPh₃ relative to NFSI, which emerged as the critical determinant in this reaction.

### Catalyst-controlled cyclization of 1,*n*-enynes

The core function of a catalyst lies in providing a new, energetically more favorable pathway for the reaction. Different catalytic systems, with their unique physical structures, chemical compositions, and electronic properties, could form distinct intermediates or transition states with reactants, thereby fundamentally altering the reaction pathway. Therefore, many research groups have made significant contributions to the divergent synthesis of small-molecule frameworks by modulating catalyst types to steer reaction pathways.

**Scheme 20:**
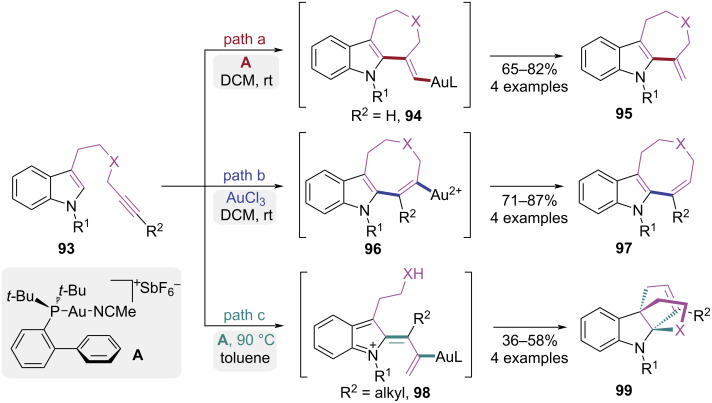
Gold-catalyzed cycloisomerization of indoles with alkynes.

In 2006, the Echavarren group reported an intramolecular gold-catalyzed cyclization of indole-alkynes, achieving control over the annulation pathway through modulation of Au oxidation state (Scheme 20) [30–31]. The seven-membered heterocycles **95** was formed via Au(I)-catalyzed 7-*exo*-dig cyclization of *N*-propargyl tryptamines **93** (Scheme 20, path a). However, the indoloazocines **97** was afforded by Au(III)-catalyzed 8-*endo*-dig cyclization via intermediated **96** (Scheme 20, path b). Interestingly, prolonged reaction time under Au(I) catalysis facilitated the formation of olefin intermediate **98**, which then underwent further Au(I)-catalyzed transformation to afford tetracyclic products **99** (Scheme 20, path c). This allenylation reaction provided efficient access to functionalized indole derivatives by regulating catalyst systems and substituent patterns.

**Scheme 21:**
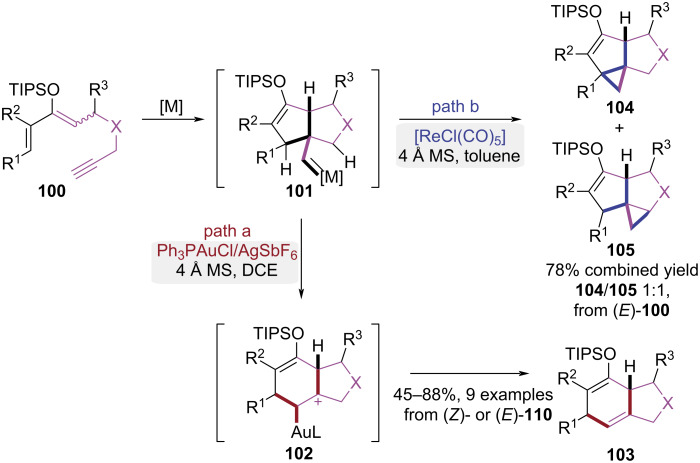
Catalyst-dependent cycloisomerization of dienol silyl ethers.

In 2010, the Iwasawa group established a stereoselective synthetic strategy toward bicyclo[4.3.0]nonane frameworks via geminal carbo-functionalization of 3-siloxy-1,3-dien-7-ynes (Scheme 21) [32]. A stereoselective sequence initiated by 5-*exo*-dig cyclization and Michael addition under cationic gold catalysis to generate strained bicyclic gold-carbene complex **101**, which was transformed to metastable intermediate **102** via stereospecific 1,2-alkyl migration. After protodemetalation, the bicyclo[4.3.0]nonane compounds **103** were obtained (Scheme 21, path a). Interestingly, the gold-catalyzed cyclization of *E*/*Z* mixture of **100** afforded bicyclo[4.3.0]nonanes **103** with configurations distinct from those generated through thermal Diels–Alder cycloaddition of (*Z*)-**100**. However, when [ReCl(CO)_5_] was used as the catalyst, a regioselective C–H bond insertion pathway was observed for substrate (*E*)-**100**, leading to the formation of tricyclic products **104** and **105** (Scheme 21, path b). This strategy employed transition metal-catalyzed carbene intermediates to mediate stereoselective cyclization, affording bicyclo[4.3.0]nonane derivatives with configurations distinct from Diels–Alder adducts.

**Scheme 22:**
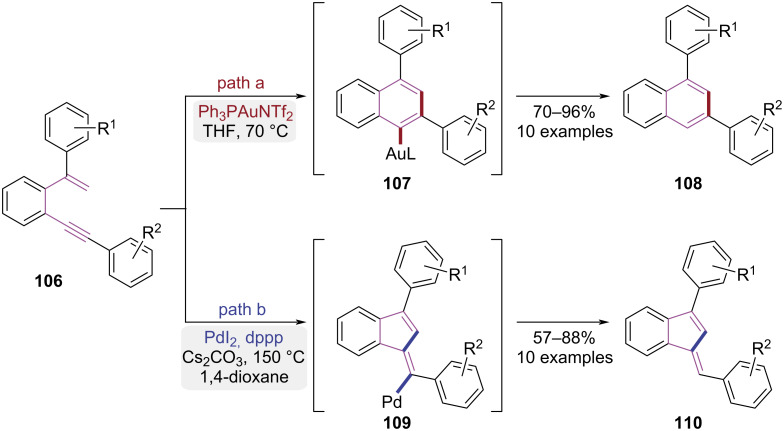
Cycloisomerization of aromatic enynes governed by catalyst.

In 2013, Alami and co-workers demonstrated catalyst-dependent cycloisomerization of aromatic enynes **106** (Scheme 22) [33]. When Ph_3_PAuNTf_2_ was employed as the catalyst, a 6-*endo-*dig cyclization occurred after the alkyne was electrophilically activated, leading to the formation of aryl naphthalene derivatives **108** (Scheme 22, path a). However, when the PdI_2_/dppp catalytic system was used, the reaction pathway changed significantly. The (*E*)-benzofulvene product **110** was exclusively generated through palladium-catalyzed C–H activation followed by a 5-*exo-*dig cyclization (Scheme 22, path b). This study established a general method for the divergent syntheses of phenylnaphthalenes and benzofulvenes from aromatic enyne precursors, where product selectivity was controlled by the catalytic system employed.

**Scheme 23:**
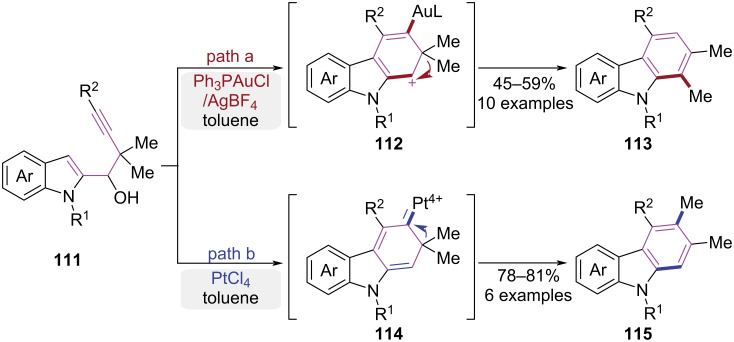
Catalyst-dependent 1,2-migration in cyclization of 1-(indol-2-yl)-3-alkyn-1-ols.

In 2014, Ma and co-workers disclosed a regioselective synthetic strategy for carbazole derivatives, where the directionality of alkyl migration was modulated by the choice of transition metal catalysts (Scheme 23) [34]. When the Ph_3_PAuCl/AgBF_4_ system was employed, the alkyne underwent an intramolecular nucleophilic attack by the C-3 position of indole to form a carbocation intermediate **112**. This intermediate underwent a Wagner–Meerwein-type 1,2-alkyl migration, ultimately leading to the construction of carbazole **113** (Scheme 23, path a). In contrast, the utilization of PtCl_4_ as a catalyst induced a distinct reaction pathway involving a platinum carbene intermediate **114**. This intermediate underwent a different 1,2-alkyl shift, leading to the selective formation of carbazole derivative **115** (Scheme 23, path b). Collectively, these findings demonstrate that the choice of transition metal catalyst critically governs the regioselectivity of 1,2-alkyl migration processes, thereby providing a strategic approach for the efficient synthesis of structurally diverse polysubstituted carbazoles.

**Scheme 24:**
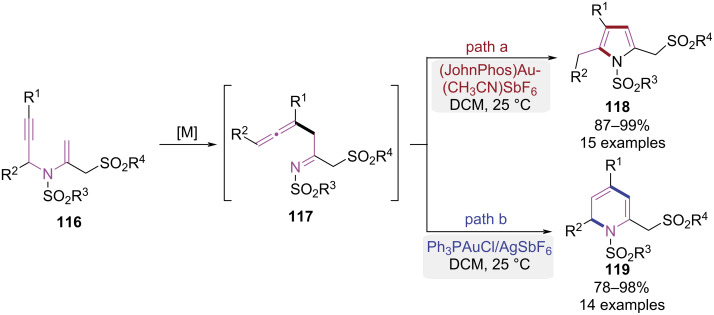
Gold-catalyzed cycloisomerization of *N*-propargyl-*N*-vinyl sulfonamides.

In 2015, Menon et al. developed a pathway-controllable gold-catalyzed cycloisomerization for the divergent syntheses of 2-sulfonylmethylpyrroles and 1,2-dihydropyridine derivatives (Scheme 24) [35]. The substrate **116** was initially transformed to a β-allene imine intermediate **117** via a gold-catalyzed propargyl-Claisen rearrangement. When the gold(I) complex [JohnPhosAu(CH_3_CN)SbF_6_] was employed, a subsequent 5-*exo-*dig cyclization followed by aromatization steps occurred, ultimately affording the 2-sulfonylmethylpyrrole **118** (Scheme 24, path a). Conversely, utilization of the Ph_3_PAuCl/AgSbF_6_ catalytic system induced a different reaction pathway. Tautomerization of intermediate **117** produced an *aza*-triene species, which underwent a 6π-*aza*-electrocyclization to afford 1,2-dihydropyridine derivatives **119** (Scheme 24, path b). This work established a divergent cycloisomerization of *N*-propargyl-*N*-vinyl sulfonamides governed by catalyst, delivering structurally distinct 2-sulfonylmethylpyrroles and dihydropyridine products with high selectivity.

**Scheme 25:**
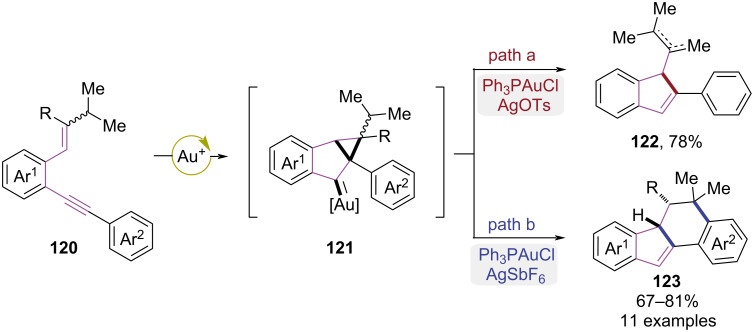
Gold(I)-mediated enantioselective cycloisomerizations of *ortho*-(alkynyl)styrenes.

In 2015, the Sanz group developed a gold(I)-catalyzed regioselective cyclization strategy leading to divergent syntheses of indenes and polycyclic compounds (Scheme 25) [36]. The substrates **120** featuring an isopropyl substituent was transformed to a carbene intermediate **121** via a gold-catalyzed 5-*endo-*dig cyclization. When the Ph_3_PAuCl/AgOTs system was used, indene derivative **122** was obtained by deprotonation (Scheme 25, path a). In contrast, 1,2-hydrogen migration was favored using the Ph_3_PAuCl/AgSbF_6_ system, ultimately resulting in the formation of dihydrobenzo[*a*]fluorenes **123** via Friedel–Crafts alkylation (Scheme 25, path b).

**Scheme 26:**
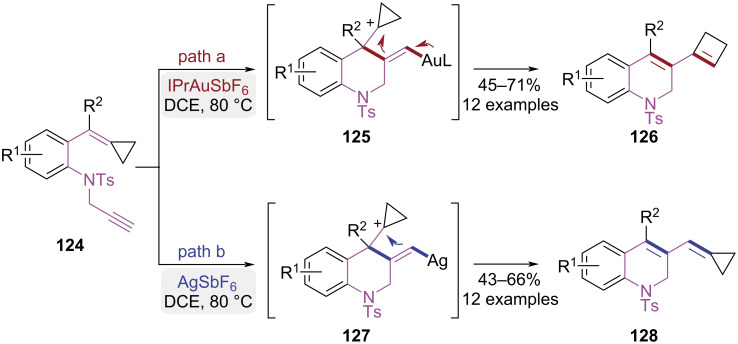
Catalyst-controlled intramolecular cyclization of 1,7-enynes.

In 2018, the Shi research group developed an innovative intramolecular cyclization strategy using 1,6-enynes as substrates for the synthesis of 1,2-dihydroquinoline derivatives (Scheme 26) [37]. Selective synthesis of 1,2-dihydroquinolines bearing cyclobutene or methylenecyclopropane frameworks was enabled by precise tuning of gold and silver catalysts. The intermediate **125** was generated via gold(I)-catalyzed nucleophilic cyclization, which underwent intramolecular rearrangement and subsequent ring expansion process to yield the product **126** (Scheme 26, path a). In contrast, a similar nucleophilic cyclization of the alkene was triggered by the silver catalyst, which preserved the intact methylenecyclopropane skeleton and yielded product **128** (Scheme 26, path b). The metal-dependent selectivity was attributed to differences in alkyne activation modes between gold and silver. The gold catalyst induced linear coordination of the alkyne to generate a carbenoid species, while silver ions favored formation of a π-activated intermediate.

**Scheme 27:**
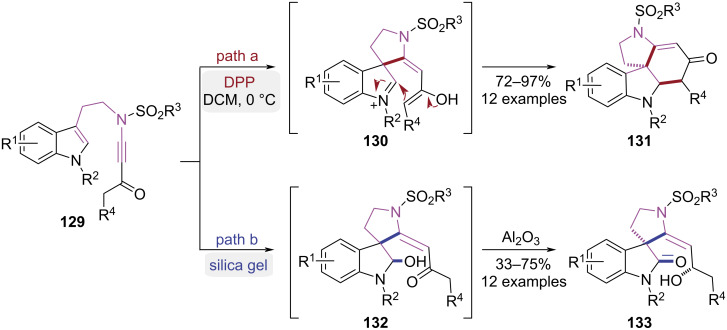
Brønsted acid-catalyzed cycloisomerizations of tryptamine ynamides.

In 2018, the Liu group demonstrated a pathway-controlled approach using tryptamine ynamides bearing Michael acceptor moieties as substrates (Scheme 27) [38–39]. Under strong Brønsted acid catalysis, protonation of the carbonyl group was achieved, which facilitated a Michael addition between the electron-rich indole C3-position and the activated ynamide. This sequence resulted in the formation of spiroindoleninium intermediate **130** (Scheme 27, path a). Subsequent intramolecular Mannich cyclization proceeded, yielding the 1*H*-pyrrolo[2,3-*d*]carbazole derivatives **131**. When silica gel was utilized as the catalytic medium, the same Michael addition happened to generate the spiroindoleninium intermediate. This transient species was subsequently trapped by water physisorbed in the silica gel, forming hemiaminal adduct **132**. A 1,5-hydride shift was then mediated by Al_2_O_3_, ultimately affording the spiro[indoline-3,3*'*-pyrrolidin]-2-one derivatives **133** (Scheme 27, path b). Through precise acid catalyst selection, the reaction pathways were strategically modulated to afford efficient construction of both 1*H*-pyrrolo[2,3-*d*]carbazole derivatives and spiro[indoline-3,3'-pyrrolidin]-2-one derivatives.

**Scheme 28:**
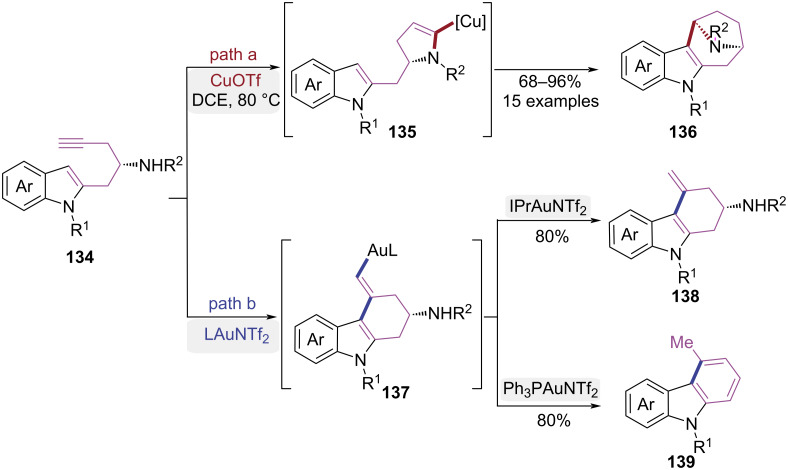
Catalyst-controlled cyclization of indolyl homopropargyl amides.

In 2019, the Ye group reported a copper-catalyzed stereospecific tandem cyclization of indolyl homopropargyl amides for the construction of bridged aza[*n*.2.1] frameworks (Scheme 28) [40]. The copper catalyst, acting as a σ,π-dual activator, induced a 5-*endo-*dig cyclization to form a vinylcopper intermediate **135**. Subsequently, a protodemetalation process first occurred, followed by an intramolecular Friedel–Crafts alkylation, ultimately resulting in the assembly of product **136** (Scheme 28, path a). Notably, a systematic screening of transition-metal catalysts revealed that structurally distinct products were obtainable from the same substrate under gold catalysis (Scheme 28, path b). When IPrAuNTf_2_ was used as the catalyst, product **138** was obtained via a 6-*exo-*dig cyclization, whereas when Ph_3_PAuNTf_2_ was employed as the catalyst, product **139** was exclusively obtained through a hydroarylation/isomerization/elimination pathway. This work disclosed an unprecedented copper-catalyzed tandem process initiated by *endo*-cyclization of indolyl homopropargyl amides, enabling atom-economical synthesis of therapeutically significant bridged aza[*n*.2.1] skeletons.

### Angle strain and configuration-controlled cyclization of 1,*n*-enynes

In cyclization reactions, angle strain and configuration exert pronounced effects on reaction pathways. These substrate-specific geometric parameters directly dictate transition state formation during ring closure. Recent advances demonstrated that deliberate manipulation of angle strain and configuration enabled divergent skeletal outcomes under identical reaction conditions.

**Scheme 29:**
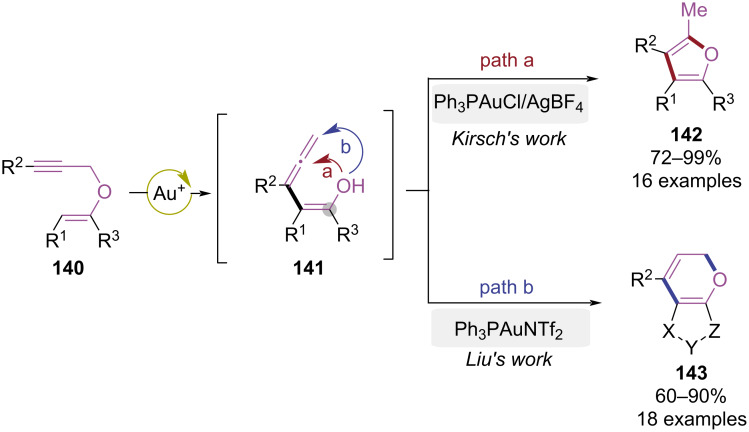
Angle strain-dominated 6-*endo*-trig cyclization of propargyl vinyl ethers.

In 2016, the Liu group achieved unconventional Au(I)-catalyzed 6-*endo*-trig cyclization by modulating the angle strain of the enol ether (Scheme 29) [41]. In previous studies [42], propargylic vinyl ethers **140** underwent Au(I)-catalyzed propargyl-Claisen rearrangement to form an allene intermediate **141**, which subsequently underwent 5-*exo*-trig cyclization to construct polysubstituted furan compounds **142**. Liu et al. discovered that the regioselectivity of cyclization could be completely altered by introducing a cyclic structure to modify the bond angle of the enol ether, exclusively yielding furopyran derivative **143** via 6-*endo*-trig cyclization. This pioneering study altered Au(I)-catalyzed 5-*exo*-trig cyclization preference through angle strain modulation, establishing an efficient strategy for the synthesis of furopyran derivatives.

**Scheme 30:**
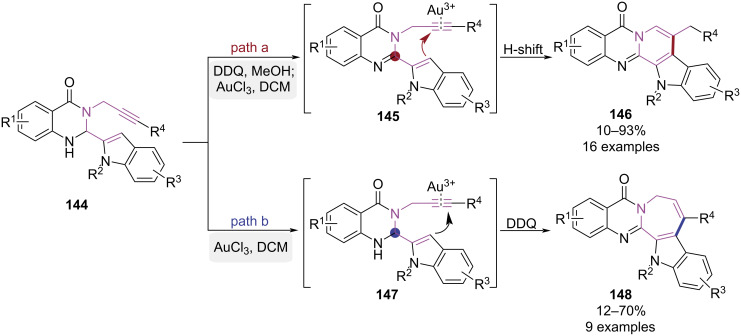
Angle strain-controlled cycloisomerization of alkyn-tethered indoles.

In 2018, Su and co-workers developed a gold-catalyzed cyclization strategy for the synthesis of 1,2- and 2,3-fused quinazolinones, which was controlled by the angle strain of a key carbon atom in the substrate (Scheme 30) [43]. Experimental results showed intramolecular cyclization depended on the hybridization state of the carbon adjacent to quinazolinone's amino group. When this carbon atom was in a sp^2^ hybridization state, the C3 position of the indole attacked the inner side of the triple bond under the action of a gold catalyst, leading to a 6-*exo-*dig cyclization to yield 1,2-fused quinazolinones **146** (Scheme 30, path a). When the carbon atom was in a sp^3^ hybridization state, the C3 position of the indole attacked the outer side of the triple bond, resulting in a 7-*endo-*dig cyclization to produce 2,3-fused quinazolinones **148** (Scheme 30, path b). This hybridization-controlled annulation strategy enabled efficient access to rutaecarpine core structures, demonstrating notable utility in the synthesis of this biologically significant alkaloid.

**Scheme 31:**
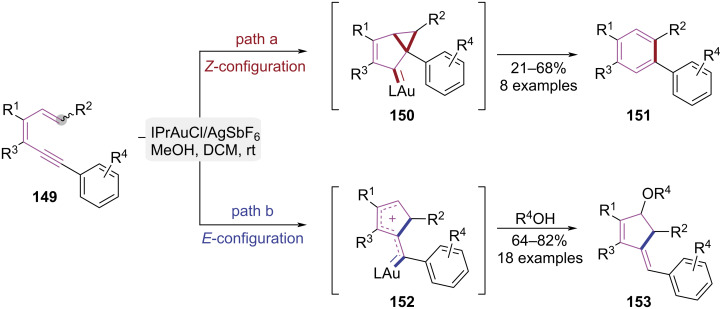
Geometrical isomeration-dependent cycloisomerization of 1,3-dien-5-ynes.

In 2020, Sanz and co-workers developed a gold(I)-catalyzed strategy achieving stereoselective construction of alkylidenecyclopentenes and benzene derivatives (Scheme 31) [44]. Notably, this study pioneered the regio- and stereoselective 5-*exo*-dig alkoxycyclization of 1,5-enynes via gold(I) catalysis. The cyclization mode was controlled by the configuration of the double bond in the substrate. In *Z*-configured substrates, steric constraints promoted 5-*endo-*dig cyclization, generating bicyclo[3.1.0]hexane intermediate **150**. Subsequent aromatization and ring expansion afforded benzene derivatives **151** (Scheme 31, path a). Conversely, *E*-configured substrates underwent gold-catalyzed alkyne activation, triggering terminal alkene 5-*exo*-dig cyclization to form carbocationic intermediate **152**. Alcohols nucleophilically trapped intermediate **152**, affording alkylidenecyclopentenes **153** with high diastereoselectivity (Scheme 31, path b). By clarifying *E*/*Z*-configuration-dependent cyclization mechanisms, this work introduced the gold(I)-catalyzed 5-*exo*-dig cyclization of 1,3-dien-5-ynes, gaining insights into regioselective control principles for gold catalysis.

### Temperature-controlled cyclization of 1,*n*-enynes

**Scheme 32:**
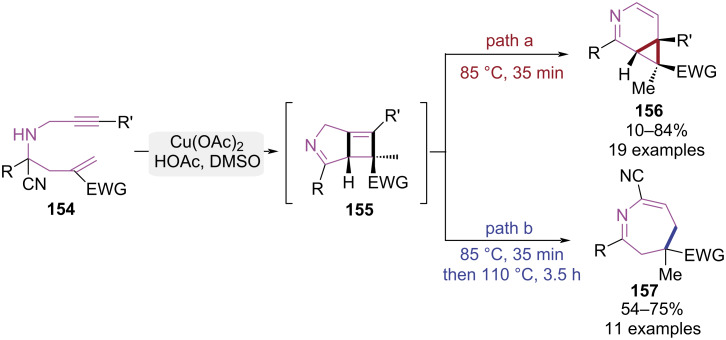
Temperature-controlled cyclization of 1,7-enynes.

Temperature, a pivotal thermodynamic parameter, not only governs reaction kinetics but also determines reaction pathways. Typically, kinetic product formation is favored at lower temperatures via pathways with reduced activation barriers, while thermodynamic products dominate under elevated thermal conditions. Recent advancements have yielded novel cyclization methodologies enabling pathway control through precise temperature regulation. In 2016, the Liao group reported a temperature-regulated strategy enabling the controlled synthesis of nitrogen-containing heterocycles via reaction pathway modulation (Scheme 32) [45]. Under catalysis of Cu(OAc)_2_ and HOAc, the substrate was subjected to decyanation and followed copper-promoted [2 + 2] cycloaddition that yielded cyclobutene intermediate **155**. Carbocation rearrangement and hydrogen elimination then occurred, affording 3-azabicyclo[4.1.0]hepta-2,4-diene derivatives **156**. In contrast, when the temperature was raised to 110 °C, intermediate **155** underwent a 6π electrocyclic ring-opening, which was trapped by in situ-generated cyanide ions to form 4,5-dihydro-3*H*-azepines **157**.

**Scheme 33:**
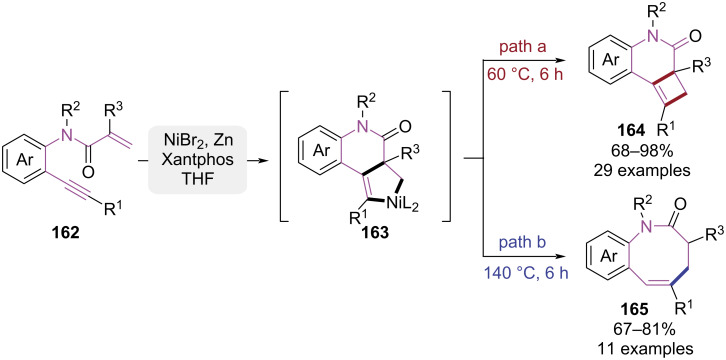
Cycloisomerizations of *n*-(*o*-ethynylaryl)acrylamides through temperature modulation.

In 2021, the Zhou group reported a nickel-catalyzed cyclization strategy using *N*-(*o*-ethynylaryl)acrylamides as substrates, achieving divergent access to dihydrocyclobuta[*c*]quinolin-3-ones and benzo[*b*]azocin-2-ones (Scheme 33) [46]. The reaction pathway was governed by thermal modulation, wherein 60 °C initiated nickel-mediated intramolecular [2 + 2] cycloaddition to form dihydrocyclobuta[*c*]quinolin-3-one framework **164**. Conversely, when the temperature was elevated to 140 °C, thermal ring-expansion of the four-membered intermediate was induced through C–C bond cleavage/reorganization, affording the eight-membered benzo[*b*]azocin-2-one product **165**. This methodology was distinguished by operational simplicity, high efficiency, and scalable synthesis. Moreover, temperature modulation achieved rapid access to cyclic compounds with distinct ring sizes.

**Scheme 34:**
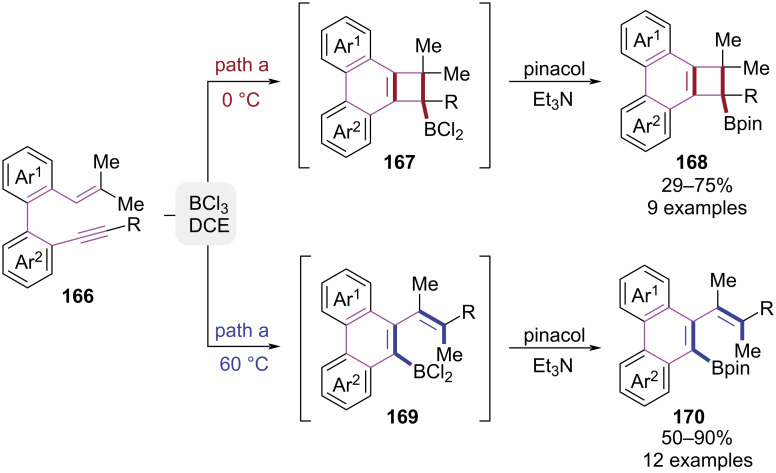
Temperature-controlled boracyclization of biphenyl-embedded 1,3,5-trien-7-ynes.

In 2022, biphenyl-embedded 1,3,5-trien-7-ynes were employed by the García group to construct borylated polycyclic products via a temperature-controlled boracyclization reaction mediated by BCl_3_ (Scheme 34) [47]. At 0 °C, BCl_3_-mediated activation of the alkyne triggered an intramolecular [2 + 2] cycloaddition, generating a boron-chlorine intermediate **167**. Subsequent treatment with triethylamine/pinacol induced ring closure, affording the phenanthreno[1,3-*b*]cyclobutane borate ester **168**. In contrast, when the temperature was elevated to 60 °C, the reaction underwent a BCl_3_-driven skeletal rearrangement involving methyl migration and alkyne cleavage to form the boronated phenanthrene framework **170**. It is worth mentioning that a unique skeleton rearrangement, supported by DFT calculations, was proposed in this work, which was unprecedented in BiCl_3_-promoted cyclization.

## Conclusion

This comprehensive review has systematically delineated the conceptual framework and innovative applications of pathway economy in the cyclization chemistry of 1,*n*-enynes. The strategic integration of pathway economy principles not only redefines reaction design paradigms but also establishes a sustainable platform for precision organic synthesis. By judiciously modulating reaction parameters – including solvent polarity, catalyst architecture, ligand electronic effects, and substrate strain effects – chemists can achieve unprecedented control over reaction pathways. This approach enables divergent access to complex molecular architectures from a unified substrate platform, as exemplified by recent breakthroughs in polycyclic skeleton construction and stereo-divergent cyclization.

The implementation of pathway economy fosters significant advancements in green chemistry by minimizing synthetic steps, reducing waste generation, and enhancing atom economy. Notably, this strategy has demonstrated remarkable potential in pharmaceutical synthesis, where the rapid generation of molecular diversity from simple precursors is paramount. Looking forward, the fusion of pathway economy with machine learning algorithms and high-throughput experimentation holds promise for accelerating reaction optimization. Furthermore, expanding this concept to other reaction manifolds – such as electrocyclic processes and photoredox catalysis – may uncover new avenues for molecular innovation. The pursuit of pathway-economical synthesis represents a paradigm shift toward sustainable and intellectually rewarding synthetic methodologies, with far-reaching implications for both fundamental science and industrial applications.

## Data Availability

Data sharing is not applicable as no new data was generated or analyzed in this study.
